# Prevalence and determinants of multidimensional frailty in hospitalized older adults with coronary heart disease: a LASSO regression analysis

**DOI:** 10.3389/fcvm.2025.1588288

**Published:** 2025-07-18

**Authors:** Ping Zhu, Dongmei Mei, Yan Yang, Defang Meng, Yaoyao Hu, Xiaoyan Wang

**Affiliations:** ^1^ Department of Cardiology, Affiliated Hospital of Jiangnan University, Wuxi, China; ^2^ Wuxi School of Medicine, Jiangnan University, Wuxi, China

**Keywords:** multidimensional frailty, prevalence, determinants, coronary heart disease, older adults, LASSO regression

## Abstract

**Purpose:**

Frailty has increasingly been recognized as a multidimensional syndrome and is particularly prevalent among older adults with cardiovascular disease. This study aimed to assess the prevalence of multidimensional frailty and identify its key determinants in hospitalized elderly patients with coronary heart disease (CHD), with the goal of informing targeted strategies for early assessment and intervention.

**Patients and methods:**

A cross-sectional study was conducted involving 508 patients aged 60 years or older who were hospitalized with CHD at a tertiary hospital in China. Frailty was assessed using the Tilburg Frailty Indicator (TFI). Variables with statistical significance in univariate analysis were entered into a Selection Operator (LASSO) regression for selection. Least Absolute Shrinkage and LASSO regression and multivariate logistic regression analyses were performed to identify significant predictors.

**Results:**

A total of 508 elderly patients with coronary heart disease who were hospitalized were included in the study, of whom 270 patients developed multidimensional frailty, resulting in an incidence rate of 53.15%. Variables with statistical significance in univariate analysis were entered into a LASSO regression for selection. Those retained by LASSO were then included in a multivariate logistic regression model. Multivariate analysis identified the following independent risk factors for multidimensional frailty: Age ≥ 75 years (OR = 2.821; 95% CI: 1.671–4.761); female (OR = 2.279; 95% CI: 1.426–3.643); insomnia (OR = 2.147; 95% CI: 1.374–3.354); depressive symptoms (OR = 4.233; 95% CI: 2.629–6.816). Conversely, higher scores on activities of daily living (ADL) (OR = 0.952; 95% CI: 0.921–0.984) and greater social support (OR = 0.935; 95% CI: 0.901–0.971) were protective against multidimensional frailty.

**Conclusion:**

Multidimensional frailty is highly prevalent in hospitalized CHD patients and independently associated with psychosocial and functional factors. Early screening and integrated interventions targeting these determinants are essential to improve clinical outcomes.

## 1 Introduction

The global aging population has led to a marked increase in the morbidity and mortality associated with cardiovascular diseases, which are now the leading cause of death worldwide. Cardiovascular diseases are responsible for approximately 32% of all global deaths, with heart disease and stroke accounting for 85% of these fatalities. Among these, coronary heart disease (CHD) remains the most common cause of death related to heart disease (1). According to the latest cardiovascular health report from China, an estimated 330 million people are affected by cardiovascular conditions, including 11.40 million individuals diagnosed with CHD. This high prevalence imposes a substantial disease burden and significant economic strain on both patients and the healthcare system (2).

In recent years, frailty has emerged as a common geriatric syndrome, characterized by a decline in physiological reserve (3), and is associated with adverse outcomes such as disability and mortality (4). Among elderly patients with coronary heart disease (CHD), the prevalence of frailty is estimated to be approximately 30% (5), Previous studies have demonstrated that frail hospitalized elderly patients with CHD experience higher rates of unplanned hospital visits and all-cause mortality compared to their non-frail counterparts, with incidence rates of 36.1% and 11.4%, respectively (6). Moreover, frailty has been identified as a significant predictor of clinical prognosis. These findings underscore the urgent need for effective frailty management strategies in hospitalized elderly patients with CHD.

Traditionally, frailty has been viewed as a physical condition; however, Gobbens (7) et al. have proposed an integrated model of frailty from the perspective of the life course, arguing that Multidimensional frailty is a dynamic state that arises from a range of factors, including age, education, income, sex, ethnicity, life circumstances, life events, genetics, and disease, affecting one or more functional domains—physical, psychological, or social—and increasing the risk of adverse health outcomes. Furthermore, the integrated model of frailty is more in line with the concepts of the modern biopsychosocial medical model and the health concept of healthy aging (8), where studies have demonstrated that multidimensional frailty increases the risk of cardiovascular disease in older adults (9). However, most of the current studies have focused on older patients with CHD and somatic frailty, ignoring other dimensions of frailty. It has been shown that habit formation interventions can slow the progression of frailty (10). Therefore, timely assessment of frailty and intervention measures can slow or reverse the development of frailty-related adverse outcomes.

Building on previous research that highlights the significant impact of frailty on outcomes in elderly patients with coronary artery disease, this study aims to assess the level of multidimensional frailty in hospitalized elderly patients. Additionally, it seeks to explore the factors influencing multidimensional frailty in these patients. The findings will provide a scientific basis for clinical practice and inform intervention strategies.

## 2 Material and methods

### 2.1 Study participants

Older patients with CHD were selected from the Department of Cardiology of the Affiliated Hospital of Jiangnan University between September 2023 and June 2024 using a convenience sampling method. Based on the required observational sample size, the total sample size was at least 5–10 times the number of independent variables (11). A total of independent variables were included in this study (note that ethnicity was not included as an independent variable, since all selected patients were Han Chinese), and taking into account a 10% loss-to-follow-up rate, the required sample size was at least 117 cases, and 508 cases were initially included in this study. Inclusion criteria: (1) had a confirmed clinical diagnosis of coronary heart disease: angiographically confirmed stenosis of ≥50% in ≥1 major coronary heart or previous revascularisation (according to ESC guidelines) (12); (2) age ≥ 60 years; (3) Voluntarily enrolled patients and signed an informed consent form and can express themselves. Exclusion criteria: (1) Patients with tuberculosis, advanced malignant tumors, HIV infection, severe chronic gastrointestinal diseases, or psychiatric disorders such as schizophrenia or depression; severe chronic gastrointestinal diseases, or psychiatric disorders such as schizophrenia or depression; (2) people who have participated in a clinical drug trial within 3 months; and (3) patients with NYHA Cardiac Function Class IV. This study was approved by the Ethics Committee of the Affiliated Hospital of Jiangnan University (approval number: LS2023085).

### 2.2 Research methods

#### 2.2.1 General information questionnaire

A general information questionnaire was designed by the researchers, the patient's age, sex, BMI, education level, mode of residence, mode of medical treatment, history of smoking, history of alcohol consumption, Insomnia, sports and exercise, cardiac function grading, multimorbidity coexisting condition (combining ≥ two chronic diseases), and history of medication taking were extracted from the cases.

#### 2.2.2 Tilburg frailty indicator (TFI)

TFI was developed by Gobbens et al. (7) in 2010 to assess multidimensional frailty in patients. The scale includes 15 items across three domains: physical, psychological, and social frailty. The total score ranges from 0 to 15, with each item scored as either “0” or “1” (13). In 2013, Xi Xing and colleagues (14) translated the scale into Chinese and validated it in elderly patients with chronic diseases. The Chinese version showed good reliability with a Cronbach's α coefficient of 0.686, making it a valid tool for assessing multidimensional frailty in elderly patients with chronic conditions in China.

#### 2.2.3 S-item geriatric depression scale (GDS-5)

GDS-5 simplified by Holy and colleagues (15), is used to assess depression in the elderly. The scale consists of five items, each scored as either “0” or “1.” The total score ranges from 0 to 5, with higher scores indicating more severe depression. A score of ≥2 indicates the presence of depressive symptoms. The scale has a Cronbach's *α* coefficient of 0.613, demonstrating its validity for depression screening in older adults.

#### 2.2.4 The activities of daily living (ADL) scale

The ADL scale, consisting of 10 items, is widely used in the Chinese elderly population (16). Higher total scores indicate better functional independence in daily living activities. The Cronbach's *α* coefficient for this scale is 0.88, showing good internal consistency.

#### 2.2.5 Social support rating scale (SSRS)

The SSRS, developed by Xiao et al. (17), contains 10 items and evaluates three dimensions of social support: objective support, subjective support, and support utilization. Each item is scored on a 1–5 scale, with higher total scores indicating more adequate social support. The internal consistency of the scale, measured by Cronbach's α coefficients, ranges from 0.825 to 0.896.

#### 2.2.6 Survey and quality control methods

Data for this study were collected using paper questionnaires administered to hospitalized elderly patients with coronary artery disease. Prior to the survey, the head nurse of the department provided training to ensure proper data collection. The researcher introduced the purpose and significance of the study, obtained their informed consent, and then conducted the survey. The completed questionnaires were retrieved on-site, and any omissions were identified and corrected immediately. Missing or incomplete questionnaires were excluded, and the data were double-checked before being entered into the database.

#### 2.2.7 Statistical methods

Data were analysed using SPSS 27.0, R 4.4.2 and R studio software. Quantitative data with a normal distribution were expressed as mean ± standard deviation, while data with a skewed distribution were presented as median (Q1, Q3). Qualitative data were reported as frequency and percentage. One-way analyses were performed using t-tests, chi-square tests, or Fisher's exact test as appropriate. Variables with a *p* < 0.05 in univariate analysis were entered into a LASSO regression model for variable selection. LASSO was implemented using the “glmnet” package in R, with 10-fold cross-validation employed to determine the optimal value of the penalty parameter lambda that minimized the mean cross-validated error. Variables with non-zero coefficients at the optimal lambda were retained for subsequent analysis. The selected variables were then entered into a multivariable logistic regression model to identify independent predictors. Prior to inclusion in the logistic regression model, multicollinearity among candidate predictors was assessed using the Variance Inflation Factor (VIF); all included variables had VIF values < 5, indicating no significant multicollinearity. Statistical significance was set at a two-sided *p* < 0.05.

## 3 Results

### 3.1 Comparison of general information between multidimensional frailty patients and non-frailty patients

A total of 508 older patients with CHD were enrolled in this study, with a mean age of 69 years. Among them, 350 were men and 158 were women. Based on the presence or absence of multidimensional frailty, 270 patients were assigned to the frail group and 238 to the non-frail group, corresponding to a frailty prevalence of 53.15%.

Univariate analysis was conducted using multidimensional frailty as the dependent variable. Statistically significant differences (*p* < 0.05) were observed between the two groups in terms of age, sex, body mass index (BMI), education level, marital status, smoking history, exercise habits, insomnia, cardiac function class, number of percutaneous coronary interventions (PCIs), grip strength, Activities of Daily LivinADL) score, Social Support Rating Scale (SSRS) score, and presence of depressive symptoms. A detailed comparison of baseline characteristics between the frail and non-frail groups is provided in Table 1.

**Table 1:** Comparison of general information between multidimensional frailty patients and non-frailty patients (*n* = 508).

Variable	Frailty Group (*n* = 270)	Non-Frailty Group (*n* = 238)	Test statistic	*p*-value
Age, years			*χ*² = 33.82	<0.001
60–74	176 (65.19%)	208 (87.39%)		
≥75	94 (34.81%)	30 (12.61%)		
Sex			*χ*² = 12.49	<0.001
Male	110 (40.74%)	48 (20.17%)		
Female	160 (59.26%)	190 (79.83%)		
BMI			*χ*² = 8.35	<0.001
<18.5	4 (1.48%)	8 (3.36%)		
18.5–24	162 (60.00%)	114 (47.90%)		
≥24	104 (38.52%)	116 (48.74%)		
Education			*χ*² = 26.88	<0.001
Uneducated	28 (10.37%)	4 (1.68%)		
Primary	72 (26.67%)	40 (16.81%)		
Middle	110 (40.74%)	128 (53.78%)		
High school and above	60 (22.22%)	66 (27.73%)		
Marital status			Fisher = 10.48	0.003
Unmarried	2 (0.74%)	2 (0.84%)		
Married	216 (80.00%)	214 (89.92%)		
Divorced or widowed	52 (19.26%)	22 (9.24%)		
Medical insurance			Fisher = 9.60	0.015
Countryside	28 (10.37%)	14 (5.88%)		
Urban residents	238 (88.15%)	212 (89.08%)		
Employee	2 (0.74%)	10 (4.20%)		
Other	2 (0.74%)	2 (0.84%)		
Falling			*χ*² = 0.08	0.776
No	236 (87.41%)	210 (88.24%)		
Yes	34 (12.59%)	28 (11.76%)		
Smoking			*χ*² = 19.41	<0.001
Never	140 (51.85%)	80 (33.61%)		
Smoking	76 (28.15%)	106 (44.54%)		
Quit smoking	54 (20.00%)	52 (21.85%)		
Drinking			*χ*² = 7.68	0.022
Never	152 (56.30%)	106 (44.54%)		
Drinking	58 (21.48%)	58 (24.37%)		
Quit drinking	60 (22.22%)	74 (31.09%)		
Exercise			*χ*² = 17.96	<0.001
Never	60 (22.22%)	38 (15.97%)		
Occasionally	128 (47.41%)	84 (35.29%)		
Often	82 (30.37%)	116 (48.74%)		
Insomnia			*χ*² = 29.45	<0.001
No	144 (53.33%)	182 (76.47%)		
Yes	126 (46.67%)	56 (23.53%)		
Cardiac Function Class			*χ*² = 22.61	<0.001
I	114 (42.22%)	144 (60.50%)		
II	120 (44.44%)	84 (35.29%)		
III	36 (13.33%)	10 (4.20%)		
PCIs			*χ*² = 15.86	0.001
0	188 (69.63%)	172 (72.27%)		
1	70 (25.93%)	38 (15.97%)		
2	10 (3.70%)	18 (7.56%)		
3	2 (0.74%)	10 (4.20%)		
Multimorbidity			*χ*² = 0.16	0.689
No	120 (44.44%)	110 (46.22%)		
Yes	150 (55.56%)	128 (53.78%)		
Number of medications			Fisher = 4.12	0.078
0	0 (0.00)	2 (0.84%)		
1–4	34 (12.59%)	20 (8.40%)		
≥5	236 (87.41%)	216 (90.76%)		
Grip strength, kg (mean ± SD)	19.46 ± 8.93	23.80 ± 10.08	Z = 5.15	<0.001
ADL score (mean ± SD)	89.85 ± 8.45	93.41 ± 7.46	Z = 5.00	<0.001
SSRS score (mean ± SD)	39.39 ± 6.55	43.37 ± 5.13	Z = 7.66	<0.001
Depressive symptoms			*χ*² = 86.25	<0.001
No	122 (45.19%)	202 (84.87%)		
Yes	148 (54.81%)	36 (15.13%)		

Data are presented as mean ± SD or *n* (%), as appropriate. *p*-values were calculated using *t*-tests, Mann–Whitney *U*-tests, chi-square tests, or Fisher's exact tests. ADL, activities of daily living; SSRS, social support rating scale; BMI, body mass index; PCI, percutaneous coronary intervention.

### 3.2 LASSO regression-based screening of multidimensional frailty influencing factors in elderly CHD patients

**Figure 1:**
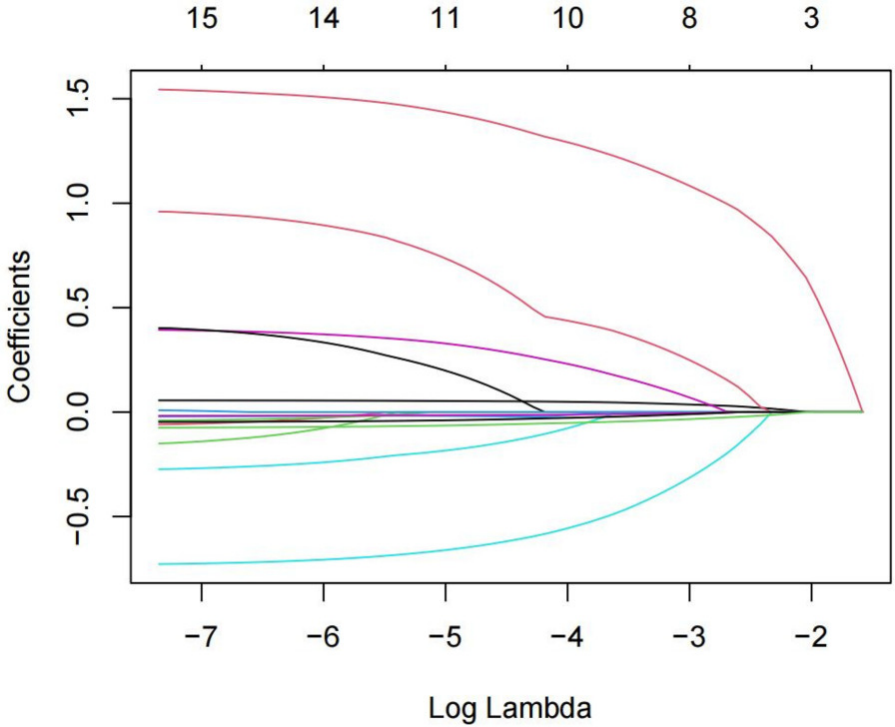
Path diagram of the coefficients of the independent variable for LASSO regression screening.

**Figure 2:**
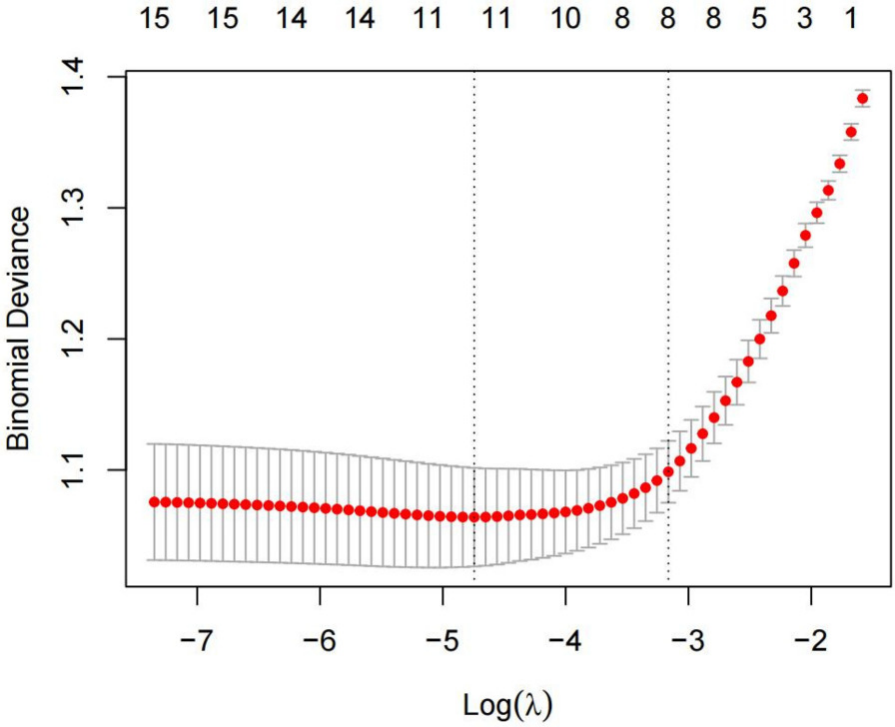
Cross-validation curve based on LASSO regression.

The 15 variables with one-factor significance (*p* < 0.05) were selected for LASSO regression analysis. Figure 1 illustrates the convergence of regression coefficients as the log(λ) parameter increases, with the coefficients approaching 0 (18); Figure 2 shows the relationship between the log(λ) values of the penalty coefficients on the *X*-axis and the likelihood bias on the *Y*-axis. A smaller *Y*-axis value indicates a better fit of the model (18). The dotted line on the left indicates the lowest MSE corresponding to the optimal tuning parameter λ (λ.min = 0.009), while the dashed line on the right represents the MSE within one standard error of λ (λ.1se = 0.042).

The model selected a λ value of 0.042 (λ.1se), which retained eight predictive variables: age, sex, cardiac function class, insomnia, grip strength, ADL score, SSRS score, and depressive symptoms. The coefficient estimates are presented in Table 2.

**Table 2:** Coefficients for categorical independent variables selected by lasso regression (*n* = 508).

Variables	Coefficient (lambda.1se = 0.042)
Age	0.039221339
Sex	0.291387173
Cardiac Function	0.101452631
Insomnia	−0.367448614
Grip strength	−0.006515809
ADL score	−0.013686798
SSRS	−0.037546747
Depressive symptoms	1.12489766

ADL, activities of daily living; SSRS, social support rating scale.

### 3.3 Multivariate logistic regression analysis of factors influencing multidimensional frailty in elderly patients with CHD

Multidimensional frailty was set as the dependent variable, and the eight variables identified through LASSO regression were included in the binary logistic regression model. Stepwise forward regression analysis was then conducted. The results revealed that age ≥ 75 years (OR = 2.354, 95% CI: 1.201–4.615), female (OR = 2.200, 95% CI: 1.137–4.255), insomnia (OR = 2.189, 95% CI: 1.167–4.105), ADL score (OR = 0.951, 95% CI: 0.907–0.998), social support score (OR = 0.932, 95% CI: 0.884–0.983), and depressive symptoms (OR = 4.324, 95% CI: 2.203–8.486) were independent risk factors for multidimensional frailty in hospitalized older adults with CHD (*p* < 0.05), as shown in Table 3.

**Table 3:** Multivariate logistic regression analysis of influencing factors of multidimensional frailty in elderly patients with CHD (*n* = 508).

Variable	β	SE	Wald	*p*-value	OR	95% CI
Age ≥75 vs. <75	1.037	0.267	15.073	<0.001	2.821	1.671–4.761
Female vs. Male	0.824	0.239	11.848	<0.001	2.279	1.426–3.643
Insomnia (Yes vs. No)	0.764	0.228	11.254	<0.001	2.147	1.374–3.354
ADL score	–0.049	0.017	8.648	0.003	0.952	0.921–0.984
SSRS score	–0.067	0.019	12.230	<0.001	0.935	0.901–0.971
Depressive symptoms	1.443	0.243	35.258	<0.001	4.233	2.629–6.816

OR, odds ratio; CI, confidence interval; ADL, activities of daily living; SSRS, social support rating scale.

## 4 Discussion

This cross-sectional study aimed to assess the prevalence of multidimensional frailty among hospitalized elderly patients with CHD and to identify the key factors associated with frailty in this population. A total of 508 elderly inpatients with CHD were evaluated. The study revealed a high prevalence of multidimensional frailty, with age, sex, insomnia, ADL, depressive symptoms, and social support identified as significant influencing factors.

The prevalence of multidimensional frailty in this cohort was found to be 53.15%, which is significantly higher than the 30.9% reported by Liu et al. (53.15% vs. 30.9%) (19). Testa et al. emphasized the significance of incorporating psychological and social factors into frailty assessments, an approach that aligns closely with the multidimensional methodology employed in our study. Furthermore, they documented a substantial burden of frailty-related factors among patients with heart failure. This finding may potentially account for the high prevalence of multidimensional frailty observed within our cohort (20).This discrepancy may be attributed to differences in the assessment tools used. Specifically, the TFI employed in this study offers a more comprehensive evaluation of frailty by encompassing multiple dimensions, including physical, psychological, and social aspects (21). In addition, the increasing aging population and the rising incidence of CHD, particularly among hospitalized patients, likely contribute to this higher prevalence. Frailty, psychological conditions, and poor family adaptation have been identified as independent risk factors for hospital readmission in elderly patients with CHD (22). Moreover, previous studies have demonstrated a bidirectional relationship between CHD and frailty. Frailty is approximately 30% more prevalent among elderly individuals with CHD compared to the general population. On one hand, frailty can exacerbate cardiac dysfunction due to diminished physical resilience. On the other hand, CHD-related hypoxia and systemic inflammation may accelerate sarcopenia and cognitive decline, further intensifying frailty severity in hospitalized patients (9, 23). These findings may help explain the high incidence of multidimensional frailty observed in this cohort. Therefore, it is essential to conduct comprehensive frailty assessments during the diagnosis and treatment of elderly CHD patients to facilitate timely management and potentially delay frailty progression, ultimately reducing the risk of adverse outcomes.

The Least Absolute Shrinkage and LASSO regression significantly enhanced our analysis by addressing key challenges associated with multicollinearity and overfitting in high-dimensional datasets. Traditional regression techniques, such as stepwise selection, often struggle with multicollinearity when managing a large number of covariates. In contrast, LASSO applies a penalty to the absolute magnitude of regression coefficients (L1 regularization), effectively shrinking less relevant variables to zero while retaining the most predictive factors (24). In this study, the tuning parameter *λ* was set to 0.042, selected through 10-fold cross-validation to achieve an optimal balance between model simplicity and predictive accuracy. This approach allowed us to reduce the initial 15 candidate variables with *p*-values < 0.05 from the univariable analysis to 8 key predictors. LASSO regression not only enhances model robustness but also minimizes bias, which is particularly important in gerontological research where variables such as social support and depression often demonstrate complex interdependencies (23).

In this study, age was identified as a significant factor influencing multidimensional frailty in hospitalized elderly patients with CHD (OR = 2.821, 95% CI: 1.671–4.761). The prevalence of multidimensional frailty was 2.354 times higher among patients aged 75 years or older compared to those under 75 years, consistent with previous findings on frailty in older adults (25, 26). Aging is associated with chronic inflammation, immune system activation, and cellular changes such as mitochondrial dysfunction, epigenetic modifications, and genomic instability—all of which contribute to the development of both CHD and physiological frailty (27). Additionally, dysregulation of the hypothalamic-pituitary-adrenal axis, along with social isolation and loneliness, frequently experienced by older adults, plays a critical role in the onset of psychosocial frailty (28). Although aging is a non-modifiable risk factor, targeted interventions that address disease management, provide psychological support, and promote social engagement can help slow the progression of multidimensional frailty and improve overall health outcomes.

Female sex was also identified as a significant factor, with older women being 2.200 times more likely to develop multidimensional frailty compared to older men (OR = 2.279, 95% CI: 1.426–3.643). A meta-analysis by Qiu et al. (29) reported a higher pooled prevalence of frailty in older women (45%, 95% CI: 39%–51%) than in older men (33%, 95% CI: 28%–39%). Several factors may contribute to this disparity. First, neuroendocrine differences, such as lower levels of insulin-like growth factor-1 and testosterone in older women, may predispose them to frailty (28). Second, immune-related variations, including higher monocyte counts and lower levels of CD56+ T cells, have been observed in women (30). Third, age-related genetic and hormonal changes, such as the decline in bone density and muscle mass, may increase susceptibility to frailty among older women (31). Finally, psychosocial influences also play a role, as women are more likely to experience chronic stress from caregiving responsibilities and are more vulnerable to gender-based violence (32). Given these factors, the prevalence of multidimensional frailty in older women warrants close monitoring. Tailored interventions should be implemented to address their specific physiological and psychosocial needs in order to mitigate frailty progression and improve health outcomes.

Insomnia was found to be an influential factor in multidimensional frailty (OR = 2.147, 95% CI = 1.374–3.354). Previous research by Fan et al. (33) demonstrated that insomnia is an independent predictor of frailty in older adults. Sleep disturbances—including difficulty initiating and maintaining sleep, early morning awakening, and daytime fatigue—are particularly prevalent in elderly individuals and are known to impair their ability to engage in physical activity and maintain social participation. These behavioral changes contribute directly to functional decline, muscle weakness, and decreased mobility, all of which are components of multidimensional frailty (34, 35). From a pathophysiological standpoint, insomnia activates the hypothalamic-pituitary-adrenal (HPA) axis and disrupts circadian cortisol rhythms, thereby increasing systemic inflammation through elevated levels of cytokines such as IL-6 and TNF-α (36). These pro-inflammatory processes accelerate muscle catabolism, endothelial dysfunction, and neurodegeneration—mechanisms that are tightly linked to both cardiovascular disease and multidimensional frailty (35, 37). Moreover, insomnia often coexists with depression and anxiety, which act synergistically to exacerbate frailty in older adults (38). Thus, early screening and non-pharmacological management of insomnia—such as cognitive behavioral therapy for insomnia (CBT-I)—should be integrated into frailty prevention strategies, particularly for older patients with CHD.

This study also found that ADL score was a protective factor for multidimensional frailty (OR = 0.952, 95% CI = 0.921–0.984), consistent with earlier research (19). ADL assessments are crucial in evaluating the functional independence of older adults, and impaired ADL performance is often linked to frailty. Older adults with frailty are at higher risk of disability, making early identification and intervention essential to prevent disability and other adverse outcomes (39, 40).

In addition, social support was found to be a protective factor against multidimensional frailty (OR = 0.935, 95% CI = 0.901–0.971). This finding is consistent with earlier research, which suggests that sufficient social support may protect older adults from developing multidimensional frailty (41). As older adults age, their functional capacity declines, and they experience changes in family structure and social relationships, leading to reduced social interactions and lower participation in social activities (42). Moreover, limited social support can lead to anxiety and depression, further exacerbating frailty (43). From a lifespan perspective, social support has been demonstrated to mitigate the adverse effects of stressors on physiological systems (e.g., the autonomic nervous system and the HPA axis) (44). Furthermore, it has been shown to reduce inflammatory markers (e.g., IL-6 and CRP) (44), we believe that social support prevents or delays the onset and progression of debilitation by reducing the burden of illness and symptoms such as anxiety and depression In addition, high levels of perceived support have been found to reduce the risk of cardiovascular disease, depression, and even all-cause mortality in older adults (44), we believe that social support prevents or delays the onset and progression of frailty by reducing the burden of illness and symptoms such as anxiety and depression (41). Thus, strengthening community-based programs, family involvement, and peer support networks may help buffer the negative effects of social isolation on health outcomes.

Finally, depressive symptoms was identified as a significant factor influencing multidimensional frailty (OR = 4.233, 95% CI = 2.629–6.816). Previous studies have shown that depression significantly increases the risk of frailty in older adults with cardiovascular disease (45). Depressive symptoms, such as low mood, cognitive decline, and social with drawal, can lead to reduced physical activity, sarcopenia, and ultimately, frailty. The shared pathophysiology between frailty and depression includes inflammation, metabolic dysregulation, and mitochondrial dysfunction, which are common in older CHD patients (46). Depressed CHD patients exhibit elevated CRP levels, reduced heart rate variability and increased oxidative stress, all of which exacerbate frailty and cardiovascular outcomes (47, 48). Therefore, comprehensive mental health screening and antidepressant therapies (e.g., SSRIs with anti-inflammatory effects) can address these common pathways and improve patient prognosis.

In summary, comprehensive frailty assessment should be an integral part of the diagnostic and therapeutic management of patients with CHD to facilitate the older identification of high-risk individuals. Multidimensional and personalized interventions are essential and should address the physiological, psychological, and social domains. Physiological Domain: Targeted strategies such as resistance and functional training can improve physical capacity and reduce age-related disability. Psychological Domain: Interventions including cognitive-behavioral therapy and individualized pharmacological treatments may help alleviate sleep disturbances and negative emotional states. Social Domain: Patients should be encouraged to participate in social activities, adopt proactive disease management behaviors, alleviate familial caregiving burdens, and promote harmonious family relationships. Furthermore, targeting chronic inflammation through the use of anti-inflammatory agents may slow the progression of frailty by modulating inflammatory pathways. These measures highlight the importance of adopting a holistic, interdisciplinary approach to frailty management in older patients with CHD (49).

However, this study has some limitations. Firstly, this cross-sectional study does not explain the causal relationship. Subsequent longitudinal studies can be conducted to discover the developmental pattern and provide a scientific basis for long-term care. Secondly, this study only included patients with coronary artery disease in the cardiology department of a tertiary hospital, which may need to be more representative of the wider population and may affect the accuracy of our data. In addition, a more extensive and diverse sample would increase the validity and generalisability of the findings.

## 5 Conclusion

Multidimensional frailty is a significant issue in hospitalized elderly patients with CHD. The main influencing factors identified in this study include age, gender, insomnia, ADL, depressive symptoms, and social support. These findings underscore the importance of healthcare professionals recognizing and addressing multidimensional frailty in this population.

Given the complex nature of frailty, it is crucial to shift from a singular assessment of physical frailty to a more comprehensive, multidimensional evaluation. Clinicians should adopt a holistic approach, considering not only the physical but also the psychological and social aspects of frailty. Comprehensive frailty management strategies are essential to delaying or even reversing the progression of multidimensional frailty, thereby improving both the prognosis and quality of life for hospitalized elderly patients with CHD.

## Data Availability

The raw data supporting the conclusions of this article will be made available by the authors, without undue reservation.
